# Analysis of Publication Interest on Preterm Birth Over Two Decades

**DOI:** 10.1007/s10995-019-02772-x

**Published:** 2019-08-02

**Authors:** Laura Visser, Marjon A. de Boer, Christianne J. M. de Groot

**Affiliations:** Department of Obstetrics and Gynecology, UMC Amsterdam, Vrije Universiteit Amsterdam, De Boelelaan 1118, 1081 HZ Amsterdam, The Netherlands

**Keywords:** Preterm birth, Preterm labour, Perinatal mortality, Publications, Bibliometric analysis, Fourth millennium development goal

## Abstract

**Introduction:**

Preterm birth (PTB) is one of the greatest challenges in obstetric and children’s healthcare. PTB remains the most important cause of perinatal morbidity and mortality worldwide. We studied the number of publications concerning PTB over the last two decades using advanced bibliometric visualization methodology. We put the number of publications in perspective of growing awareness of PTB as a major health problem.

**Methods:**

We analyzed publications over time and performed bibliometric analysis of publications on PTB in the Web of Science from 1997 to 2016. The subjects of publications were visualized using a term map showing the relevant terms occurring in titles and abstracts.

**Results:**

We identified 47,811 publications. The annual number of publications on PTB increased significantly by 443% in 2016 (n = 5027) compared to 1997 (n = 1135). *Obstetrics & Gynecology* is the leading research field (with 10.4% on the subject PTB in 2016) followed closely by *Pediatrics* (7.6% on the subject PTB in 2016), within the field *Public, Environmental & Occupational Health* minimal increase was observed (only 1% was published on the subject PTB in 2016). The publications on PTB have increased at higher rates than the overall increase of publications. In recent years we found more publications on PTB describing epidemiology and clinical characteristics/outcomes whereas in earlier years publications focused more on translational, basic research.

**Conclusion:**

A significant increase in research concerning PTB was observed over the last two decades. This increase of publications is in line with the growing global awareness of the need to reduce PTB by clinical research.

**Electronic supplementary material:**

The online version of this article (10.1007/s10995-019-02772-x) contains supplementary material, which is available to authorized users.

## Significance

*What is already known on this subject?* Preterm birth is the most important cause of perinatal morbidity and mortality worldwide. The attention for the general problem of preterm birth increased over the last decades. Yet, the incidence of preterm birth has hardly changed.

*What this study adds?* Medical research is the foundation needed to build and improve health care. A significant increase in research concerning preterm birth was observed over the last two decades. The number of preterm birth publications has increased at a much higher rate than the overall number of publications. This is in line with the growing global awareness of the need to reduce preterm birth by clinical research.

## Introduction

Preterm birth (birth before 37 weeks of gestation) is one of the most comprehensive obstetric and pediatric challenges in medical practice today. Preterm birth is the most important cause of perinatal morbidity and mortality (Ananth and Vintzileos 2006; Goldenberg et al. 2008; WHO 2016 [last reviewed november 2016]). Each year 15 million children are born prematurely, more than 1.1 million of them die due to the consequences of prematurity (Howson et al. 2012). Preterm birth is the largest cause of mortality in the neonatal period and is the largest contributor to all deaths of children under 5 years (Liu et al. 2016). The prevalence of preterm birth varies strongly around the world (5–18%) (Blencowe et al. 2012).

The attention for the general problem of preterm birth increased over the last decades (Bhutta et al. 2014). In 2000 eight international development goals for the year 2015 were formulated by the United Nations: the Millennium Development Goals. The 4th Development Goal was to reduce infant mortality by two/thirds in 2015 (Assembly 2000).

Compared to the year 1990 a reduction of 53% of the under-5-year-old mortality rate was realized by 2015 (Assembly 2000; You et al. 2015). This improvement is due to improvement of obstetric and neonatal care while the incidence of preterm birth has hardly changed. Despite this remarkable progress in the improvement of child survival, the 4th Millennium Goal of a two/thirds reduction of under-5-year-old mortality rate has not yet been achieved. The biggest barrier seems to be the inability to reduce number of neonatal deaths and deaths of prematurity (Lawn et al. 2010). Consequently, preterm birth is the leading cause of death under 5 years of age in 2015 (Liu et al. 2016). Therefore a new set of goals for future international development were formulated in 2015; the Sustainable Development Goals to be reached in 2030 (UN 2015).

Medical research is the foundation needed to build and improve a rational health care system. We performed a bibliometric analysis to understand any impact of societal developments, reflected by the international Development Goals formulated by the WHO, on medical research. We evaluated the amount of publications on the subject of preterm birth and we used an advanced bibliometric visualization methodology to show the main research topics studied in the literature on preterm birth and the scientific development over time.

## Methods

We identified publications related to preterm birth in the Web of Science (WoS) bibliographic database. We selected all publications in the WoS database in the period 1997–2016 that include ‘preterm birth’, ‘preterm labo(u)r’, ‘preterm delivery’ or ‘prematurity’ in their title, abstract or keywords. The WoS database classifies all publications into a document type, such as ‘article’, ‘review’, ‘meeting abstract’, ‘letter’, or ‘editorial material’. In order to select only the most significant publications we excluded all publications but ‘article’ or ‘review’. Hence, we selected 47,811 publications to include in our analysis. We collected the title, abstract, and publication year for each publication.

The WoS database assigns one or more fields to each publication; this field is based on the publishing journal. In case a publication belongs to more than one field, the publication counts fractionally in each of the fields to which they belong. For instance, a publication belonging to two fields is counted as half a publication in each of the two fields. Publications in general medical journals, such as the *Lancet* and the *British Medical Journal*, are assigned to a special field called General & Internal Medicine. In this way for each publication the field to which the publication belongs in the WoS database was determined.

To evaluate the validity of the selected publications, three random samples of 100 publications (from the years 1997, 2006 and 2016) were analyzed by reading (LV) their titles and abstracts. 92% of the first sample, 99% of the second and 88% of the third sample turned out to concern preterm birth.

The subsequent phase was performed using the VOSviewer software (freely available at www.vosviewer.com), a tool for constructing and visualizing bibliometric networks (van Eck and Waltman 2010b). We used this software to conduct the linguistic analysis of the selected publications (Rodrigues et al. 2014). In order to visualize the structure of research concerning ‘preterm birth’ we created a term map utilizing the VOSviewer software (Van Eck and Waltman 2014). A term map is a distance based, two-dimensional representation of a research field, the distance between two terms approximately indicates the relatedness of the terms; strongly related terms are located close to each other and less strongly related terms are located further away from each other. Different areas in a term map represent research areas; colors are used to visualize the different areas or to indicate differences between areas in the changes in publication output over time.

In the analysis the titles and abstracts of the 47,811 publications were parsed by VOSviewer, using natural language processing techniques. A list of 459,458 noun phrases that occur in these publications was generated. We removed all noun phrases occurring in fewer than 75 publications resulting in 3750 terms, this way we eliminated insignificant noun phrases, which occur in only a small number of publications. Subsequently, 60% of most relevant terms were selected resulting in 2250 terms; these are the terms referred to in this article. This cut-off limit was used because this limit is the default cut-off limit suggested by the software resulting in a selection of the most relevant terms. An additional 222 irrelevant terms such as terms referring to countries/languages, general scientific concepts or weight measurements were manually removed.

The relevance of the noun phrases, to which we will from here on reference to as terms, was determined using a computer algorithm(van Eck and Waltman 2011) incorporated in the VOSviewer software, this algorithm aims to filter out general terms such as ‘method’, ‘result’, and ‘study’.

In the following step, the number of publications in which the terms co-occurred was determined. Two terms co-occur if they both occur at least once in the title or abstract of the publication. The larger the number of publications in which two terms co-occur, the stronger the terms can be considered to be related to each other. The term co-occurrence frequencies served as input for the VOS mapping technique (van Eck and Waltman 2010a). Using this technique a location in a two-dimensional space was determined for each term. The VOS mapping technique aims to position strongly related terms close to each other in a two-dimensional space and terms that do not have a strong relation further away from each other. A clustering technique (Waltman 2010) was used to assign each term to a cluster, this technique is available in the VOSviewer software. This clustering technique is strongly related to modularity based clustering which is used for clustering objects in a network (Newman 2004). Terms that are assigned to the same cluster tend to be strongly related to each other, while terms assigned to different clusters usually do not have a strong relation. In the final step an age score was calculated for each term, this score equals the average publication year of all preterm birth publications in which the term occurs (in the title or abstract).

The age scores of terms can be visualized using colors. We used colors that range from blue (score of 2007 or lower) to green (score of 2009) to yellow (score of 2013 or higher). Therefore, if a term is colored blue, the term occurs mainly in older publications. On the other hand, if a term is colored yellow, the term occurs mainly in more recent publications.

The interpretation of the term maps has been described in earlier publications (de Groot et al. 2015) and can be summarized as follows:

- Each circle represents a term. Sometimes no label is shown for a term, more labels become visible by using the VOSviewer software to zoom in into a specific area in a term map.
- The size of each term is directly proportional to the number of publications in which the term occurs.
- The distance between two terms reflects their relatedness, as measured by their co-occurrence frequency.
- The horizontal and vertical axes have no special meaning in a term map, rotation does not affect its interpretation.
- The color of a term indicates either the cluster to which the term has been assigned or the age score of the term.
- This study is not based upon clinical study or patient data.

## Results

### Analysis by Research Area

**Fig. 1:**
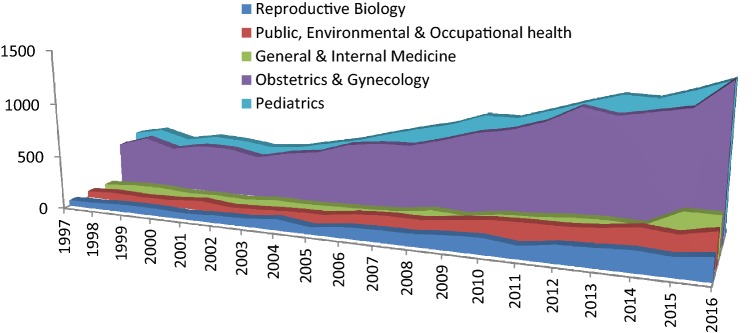
Increase of amount of publications per year on the subject of preterm birth

**Fig. 2:**
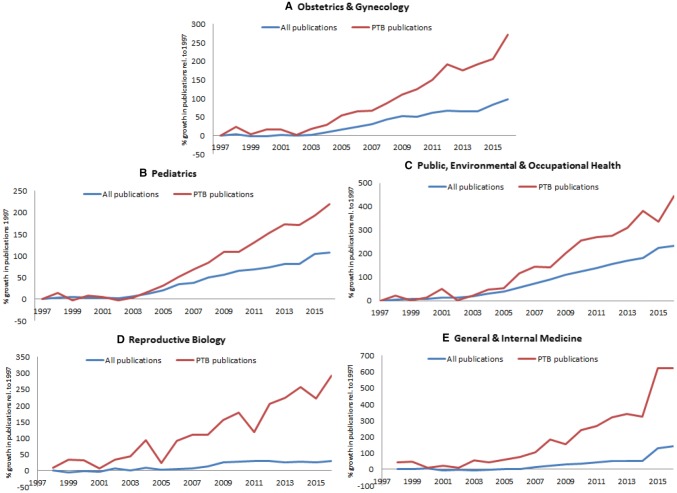
Growth in number of publications on the subject of preterm birth compared to the overall growth of publications in the five most significant research areas. **a** shows the field of ‘Obstetrics and gynecology’. **b** shows the field of ‘Pediatrics’. **c** shows the field of ‘Public, Environmental & Occupational Health’. **d** shows the field of ‘Reproductive Biology’. **e** shows the field of ‘General & Internal Medicine’

47,811 publications on the subject of preterm birth were identified. A considerable increase of the yearly publication rate on the subject of preterm birth was observed, from 1135 publications in 1997 to 5027 publications in 2016, an increase of 443%. The research mainly took place in the field of ‘*Obstetrics and Gynecology*’ followed by ‘*Pediatrics*’, ‘*Public, Environmental & occupational health`,General & Internal Medicine*’ and ‘*`Reproductive biology`*’. The increase in the amount of publications, broken down by scientific field, can be found in Fig. 1. This figure shows a steepening of the increase from 2003 onwards. The growth of publications on preterm birth in the five most significant fields were compared to the overall growth of publications in each specific field and are presented separately in Fig. 2a–e.

The field of *Obstetrics & Gynecology* showed a steep increase. In 1997, 5.5% (389) of all 7047 publications concerned preterm birth, this portion rose to 10.4% (1445) of all 13,899 publications in 2016. All other fields show a more or less equal increase. In the field of *Pediatrics* 5.0% (440) of all 8828 publications concerned preterm birth in 1997 and this portion rose to 7.6% (1399) of all 18,332 publications in 2016. The field of *Public, Environmental & Occupational Health* showed an increase from 0.6% (61) of all 10,042 publications on the subject of preterm birth in 1997 to 1.0% (332) of all 33,399 publications by 2016. The portion of preterm birth publications in field of *Reproductive Biology* rose from 1.5% (55) of all 3576 publications in 1997 to 4.6% (215) of all 4662 publications in 2016. And finally, in the field of *Medicine General & Internal* an increase from 0.3% (52) of 18,230 publications in 1997 to 0.9% (375) of 44,028 publications in 2016 concerned preterm birth. The share of preterm birth publications has more than doubled in nearly all fields, *Reproductive Biology* has relatively the biggest share increase.

The number of publications on preterm birth has increased, far more than the overall number of publications. The overall increase is most noticeable from 2003 onwards.

### Analysis Using Term Maps

**Fig. 3:**
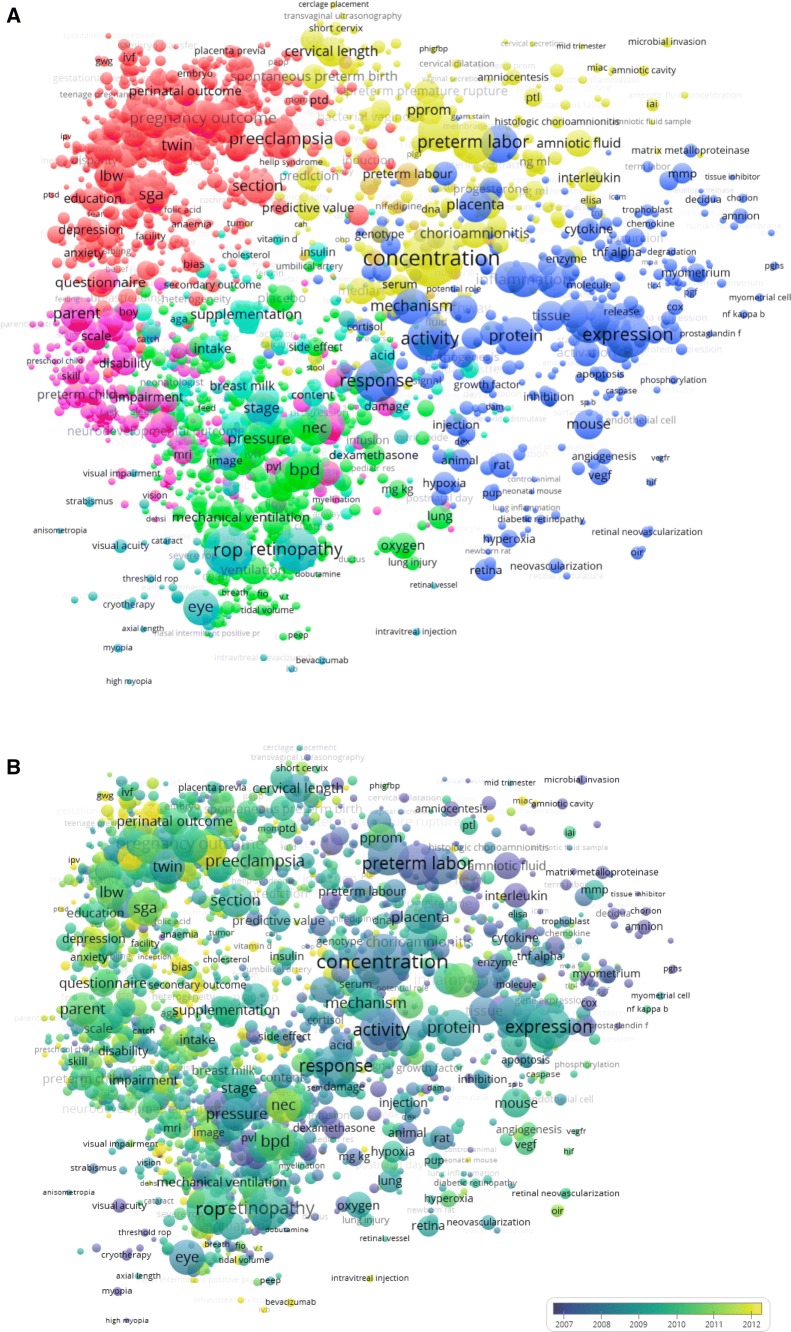
a Term map (network visualization) based on 47,811 preterm birth publications in the period 1997–2016. **b** Term map (overlay visualization) based on 47,811 preterm birth publications in the period 1997–2016

Figure 3a presents the term map based on the 47,811 publications on preterm birth in the period 1997–2016. Five clusters were identified. An interactive version of the map is available on http://www.……... The interactive map offers zooming functionality that allows the map to be explored in much more detail than its static versions in Fig. 3a (the digital map requires a system with Java support).

The clusters represent the following research areas:

- *Yellow cluster* represents cause and prevention of preterm birth with terms such as*: ‘concentration’, ‘preterm labour’, ‘preterm premature rupture’, ‘bacterial vaginosis’ and ‘cervical length’.*
- *Red cluster* represents obstetric conditions leading to preterm birth, with terms such as: *‘preeclampsia’, ‘twin’, ‘pregnancy outcome’* and *‘sga’.*
- *Purple cluster* represents clinical long term outcome, with terms such as: ‘*neurodevelopmental outcome’, ‘preterm child’, ‘behavior’, ‘brain’* and *‘injury’.*
- *Green cluster* represents pediatric, neonatal outcome, with terms such as: *‘bpd’, ‘nec’* and *‘chronic lung disease’*
- *Orange cluster* represents ophthalmology, with terms such as: *‘rop’, ‘eye’ ‘retinopathy’, ‘stage’* and ‘*laser treatment’*
- *Blue cluster* represents basic, translational research, including animal studies, with terms such as *‘inflammation’, ‘protein’, ‘placenta’, ‘cell’* and *‘expression’*

Figure 3b shows the trends over time, the clustering and representation is identical, the coloring is different. The color in this map represents the `age-score` of a term. This age-score indicates the average publication year of all preterm birth publications in which the term occurs.

Showing that in recent years emphasis was on epidemiology and clinical characteristics and outcomes. In previous years publications focused more on basic and translational research.

## Comments

Over the last two decades a strong increase of the number of preterm birth publications was observed, especially since 2003. The two most dominant research areas are *Obstetrics & Gynecology* and *Pediatrics*. The share of preterm birth publications has more than doubled in nearly all fields; *Reproductive Biology* shows relatively the biggest share increase. In all research fields the number of preterm birth publications has increased at a much higher rate than the overall number of publications. Over time we observed a shift in attention from basic and translational research to epidemiology and publications focusing on clinical characteristics and outcomes in the most recent years.

### Strengths and Limitations

An important strength of our study is its advanced bibliometric methodology. We evaluated publications not only at the level of the pre-defined fields in the WoS database, but we also used advanced bibliometric visualizations to study the structure and development of the preterm birth literature (van Eck et al. 2013).

A limitation of de selection of included publications is that some of the included publications do not cover the subject of preterm birth, this limitation could have been avoided by reading all titles and abstracts however due to the magnitude of the number of the studied publications, reading all abstracts was not realistic. Additionally, some publications may have been missed due to issues related to the coverage of the WoS database. A portion of local journals is for example not covered in the WoS database. To some degree, this problem could be solved by using multiple databases, such as WoS and Medline, in a combined fashion (van Leeuwen 2001). Publications may also have been missed because they do not contain ‘preterm birth’, ‘preterm delivery’, ‘preterm labo(u)r’ or ‘prematurity’ in their title or topic even though they do in some way relate to the topic of preterm birth.

The Millennium Goals formulated by the WHO were considered to be a reflection of the societal awareness for the problem of preterm birth. Other societal developments were not studied separately; this could be considered a limitation of this study.

### Interpretation in Light of Reduction of Preterm Birth

Our analysis shows an increasing interest in the subject of preterm birth distributed over all research fields. This increasing scientific interest is in line with the increased societal interest in reducing preterm birth, reflected in the Millennium Goals. The increase in publication rate is steady since 2003, it seems likely that this increase is largely due to the international recognition of preterm birth as a major health problem, also reflected in the 4th Millennium Goal. The biggest barrier to progress on this Goal is the inability to significantly reduce the incidence of preterm birth. In developed countries neonatal deaths related to preterm birth have been reduced due to improvements in (advanced) neonatal care. In developing countries, where women have limited access to prenatal and delivery care, the number of neonatal deaths and deaths related to preterm birth remain high. The differences between the developed and developing worlds were similarly described regarding the 5th millennium Goal; reduction of maternal mortality (de Groot et al. 2015).

In order to reach the Sustainable Development Goals by 2030, a lot of work still needs to be done and preterm birth remains a global challenge. The field *Public, Environmental & Occupational Health* has a minimal part in the research on PTB, only 1% published on the subject and therefore a potential opportunity from a societal perspective lies in this field, especially in developing countries.

### Implications for Research

(Medical) research is important to help identify new approaches in treatment and technologies in order to reduce preterm birth and reach the Sustainable Development Goals by 2030. However, the significant increase in the number of scientific publications concerning preterm birth has had little to no effect on the incidence of preterm birth. Our data showed a shift from basic research to epidemiology and clinical research. We observed a recent interest in key terms such as preeclampsia and perinatal outcomes with a potential to improve preterm birth rates and outcomes, especially when placed in the context of the *Public, Environmental & Occupational Health* domain.

In this perspective the research challenges for the developed and developing world are not equal. The developed world should lead the way with innovations in research focused on etiology, prevention and treatment. Whereas the developing world might benefit more from pragmatic research focused on addressing public health issues and implementing proven interventions.

## Conclusions

Preterm birth is the leading cause for mortality for children under 5 years. Despite the significant increase in scientific publications on the subject, the incidence of preterm birth remains unchanged. We observed an increase of 443% in the number of preterm birth publications, this is in line with the increasing global awareness of the need to reduce preterm birth. In all research fields the number of preterm birth publications has increased at a much higher rate than the overall number of publications. *Obstetrics & Gynecology* is the leading research field followed closely by *Pediatrics*.

## Electronic supplementary material

Below is the link to the electronic supplementary material.

Supplementary material 1 (TXT 153 kb)
